# Impact of different mating ratios of broiler breeder on reproductive performance during post moult phase

**DOI:** 10.1080/10495398.2024.2398707

**Published:** 2024-09-02

**Authors:** Mohammad A. Elsagheer, Omar A. Mohamed, Ibrahim M. Khattab, Marian K. Wadea

**Affiliations:** a Animal Production Department, Faculty of Agriculture, Azhar University, Assiut, Egypt; b Department of Poultry Production, Faculty of Desert and Environmental Agriculture, Matrouh University, Matrouh, Egypt; c Department of Animal and Fish Production, Faculty of Desert and Environmental Agriculture, Matrouh University, Matrouh, Egypt; d Animal and Poultry Production Department, Faculty of Agriculture, Beni Suef University, Beni Suef, Egypt

**Keywords:** Mating ratio, broiler, forced molting, fertility, hatching, embryonic mortality rate

## Abstract

Studies comparing mating ratio after forced molting are important for improving the efficiency of broiler breeder flocks. This study examined the effect of mating ratios on Egg production, chick weight, fertility, hatchability and embryonic mortality rate in Arbor Acres Plus broiler breeders post-moult phase. A total of 195 birds (62 weeks old), consisting of 15 cocks and 180 hens were randomly allocated with uniform body weight in a Completely Randomized Design into three groups based on the mating ratio (cock to hen). The groups included ratios of 1:10, 1:12, and 1:14, with each group replicated 5 times. Eggs were collected twice a day, and stored for 7 days at room temperature before placing them in the incubator. Results showed that mating ratios 1:10 and 1:12 had a positive effect on fertility rate and total hatchability compared to the 1:14 ratio. However, mating ratios did not affect laying rate, egg weight, egg mass, chick weight, hatchability of fertile eggs, and embryonic mortality rate. In conclusion, to reduce the cost of raising and caring cocks, a mating ratio of one cock to every twelve hens can be used for broiler breeders after forced molting to obtain the best fertility and hatching results.

## Introduction

Poultry production has developed significantly, transforming from a basic industry to a highly integrated sector with global competitiveness. It is one of the main pillars of the food production sector and one of the fastest-growing sectors worldwide. This development signifies its increasing importance in food production and enhancing food security.^1^

Comparative mating ratio trials are crucial to improving broiler breeder flocks’ breeding productivity. Many studies of poultry species have shown that achieving the ideal mating ratio is essential for improving productive and reproductive traits, and any imbalance may lead to adverse results.^2–4^ For example, research by Deeming and Wadland^5^ showed that pheasants mated at a cock -to-8-hens ratio showed superiority in egg production, fertility, and hatchability compared to those mated at a cock:12 hens ratio. This result is supported by findings in Japanese quails, where it was observed that the mating ratio significantly affected fertility and hatchability^6^, emphasizing the importance of managing the mating ratio. The adjustments in mating ratio significantly impact reproductive performance and can lead to either improvements or declines in reproductive traits.^7^

Although results showed the importance of mating ratios in reproductive traits, their effects may be complicated and unclear in different poultry species and breeds, and it is difficult to judge the ideal ratio and generalize the results. For example, Iyasere et al.^8^ reported that mating ratios (cock to 3 hens, cock to 6 hens, and cock to 9 hens) had no significant differences in egg production, fertility, and hatchability in native chickens. Conversely, Haghighi et al.^9^ observed significant differences in egg production on Ross 308 broiler breeders raised under different mating ratios (cock: 13.3hens, cock: 11.6hens, and cock: 10.5hens). This variability requires further studies to understand how mating ratios influence reproductive performance, to achieve optimal results.

Molting is a natural physiological cycle in birds when they rid of their old feathers and replace them with new ones, this process is followed by reproductive quiescence leading to a decrease in egg production.^10^ According to Sharma and Gupta^11^, forced molting is an effective method economically for breeders in developing nations. However, after molting, there is a noticeable decline in reproductive characteristics.^12^ Therefore, studying the effect of changing mating ratio after molting may be an important strategy for improving reproductive performance, because increasing cocks to hens may improve the chances of fertilization. On the other hand, the excessive presence of males may lead to increased aggressive behavior, which negatively affects reproductive performance. Currently, to the best of our knowledge, no earlier studies addressed the suitable mating ratio for broiler breeders post forced molting. We hypothesize that different mating ratios can affect the overall reproductive performance of broiler breeders following forced molting. Therefore, this study aims to investigate the effects of different mating ratios on egg prodction, chick weight, fertility, hatchability and embryonic mortality rate of Arbor Acres Plus broiler breeders post forced molting.

## Materials and methods

This study was conducted on a commercial farm in Minia governorate, Egypt from December 2022 to March 2023. All animal experimental procedures in this study were approved by the Animal Care and Use Committee of Alexandria University (Approval no. 0306666).

### Experiment design

A total number of 180 hens and 15 cocks, 62 weeks old with an average body weight of 3883 ± 0.27 g for cocks and 2731 ± 0.41 g for hens in the post-molt phase were selected as Arbor Acres Plus broiler breeder. Birds were induced to forced molting with ZnO in the diet at a level of 3000 mg/kg with a moderate decrease in lighting schedule from 16 to 8 h and feed restriction at 50 g/bird/day according to El-Deek and Al-Harthi^13^ and Elsagheer et al.^14^ The molting phase continued for 6 weeks until a 27% reduction in their body weight was achieved. After completion of molting, birds were divided into three groups based on their uniform body weight in a Completely Randomized Design. They were divided according to a mating ratio (cock to hen) which included ratios 1:10, 1:12, and 1:14 with 5 replications in each group. The trial lasted for 12 weeks.

### Birds housing

Birds were raised in a closed-house system in floor pens (1.5 × 1.25m) under hygienic conditions with artificial lighting, automatic ventilation, and electrical heaters to provide an inside temperature set at 22 °C and relative humidity ranging between 55% to 65%.

### Experimental diets

A basal diet was formulated to fulfill nutrient requirements according to NRC^15^ and offered in restricted amounts (162 g/cock/d and 173 g//hen/d). The basal diet for cocks had 12.07% crude protein, 3.39% fat, 6.10% crude fiber, 0.37% available phosphorus, 0.95% calcium, and 11.29 MJ/kg metabolic energy. The basal diet for hens had 13.30% crude protein, 2.96% fat, 3.16% crude fiber, 0.39% available phosphorus, 3.51% calcium, and 11.69 MJ/kg metabolic energy.

### Studied traits

#### Egg production (EP)

The eggs were collected twice daily, at 9 am and 4 pm, to calculate egg number. The laying rate (%) = (egg number/hen number) × 100. Each egg was weighed and recorded daily to calculate the average egg weight (g) and mass (g). The egg mass was calculated using the formula: (egg production × egg weight)/100.

#### Hatchability traits

Eggs were collected from each replicate at the 4th, 8th, and 12th weeks, with 50 eggs collected from each replicate. The collected eggs were stored for 7 days at 15–18 °C and 70–75% relative humidity before incubation. The standard incubation conditions were maintained from day 1 to 18 at 37.8 °C and a relative humidity of 55–60%. During the last three days of incubation, the conditions were adjusted to 36.8 °C and a relative humidity of 60–65%. The incubation was performed by using an automatic Patersime BioStreamer incubator. Eggs were candled on days 7 and 18 of incubation to determine infertile eggs and eggs with dead embryos. At the end of the incubation period, all the unhatched eggs were broken to calculate fertility, hatchability and embryonic mortality percentages. Fertility rate was calculated by dividing the number of fertile eggs by the total number of eggs produced. Total hatchability was calculated as the number of hatched eggs divided by the number of eggs set in the incubator. The hatchability of fertile eggs was calculated as the number of hatched eggs divided by the number of fertile eggs. Total embryonic mortality rate (%), mortality on days 7 (early embryonic mortality rate (%)) and day 18 (late embryonic mortality rate(%)) of incubation were determined. Chicks were weighed by an electronic balance (±0.1 g, Zhejiang Wonheng Industry And Trade Co., Ltd., China) after hatching to calculate the average chick weight (g).

### Statistical analysis

Data were statistically analyzed by the completely randomized design using the general linear model using SAS.^16^ The effect of the mating ratio (3 treatments) was treated as a fixed effect. The data were analyzed using the following statistical model:

Yij =μ+Ti +Eij

where, Yij = an observation; μ = general mean; Ti = fixed effect of ith mating ratio; Eij = error of the model, which included all the other effects not specified in the mixed model.

Differences among means of the experimental groups were determined by Fisher’s LSD test at a 5% significance level.

## Results and discussion

### Egg production

The results of a study on the effect of mating ratios for broiler breeders post molting on egg production traits and chick weight are presented in Table 1. The study found no significant difference in laying rate percentage (*p* = 0.753), egg weight (*p* = 0.605), egg mass (*p* = 0.995), and chick weight (*p* = 0.792) for broiler breeders post molting with cock: hen ratios of 1:10, 1:12 and 1:14. This suggests that egg production traits were not affected by the mating ratios used, but it provides insight into the productive performance after molting.

**Table 1.** Effect of mating ratios on egg production traits and chick weight for broiler breeders post molting.

Item	Mating ratios (cock to hen)			SEM	*p*-value
	1:10	1:12	1:14		
Laying rate, g	65.81	65.64	66.08	1.43	0.753
Egg weight, g	68.19	68.29	67.83	0.91	0.605
Egg mass, g/bird/day	44.87	44.82	44.83	0.86	0.995
Chick weight, g	46.28	46.29	45.94	0.87	0.792

SEM: standard error of the mean.

On the other hand, there is limited knowledge about the effect of mating ratio on the weight of hatched chicks in laying hens. Tona et al.^17^ found a positive correlation between hatching weight and egg weight. Additionally, Ugurlu et al.^18^ found no significant difference in hatched chick weight due to the mating ratio effect in pheasants. Therefore, the similar hatching weight of chicks found in this study could be due to the similarity in the weight of the eggs set.

### Egg fertility

The effect of mating ratios for broiler breeders post molting on egg fertility is shown in Table 2. The study found that egg fertility was significantly (*p* = 0.037) higher when the mating ratio was 1:10 and 1:12 compared to a ratio of 1:14. The egg fertility with a 1:10 and 1:12 exceeded the fertility with a 1:14 by 2.09% and 1.79%, respectively. These results suggest that lower mating ratios (1:10 and 1:12) decrease the likelihood of females not mating and may increase the frequency of males mating with the same female, leading to higher fertility levels and reduced competition between males.

**Table 2.** Effect of mating ratios on fertility rate, hatchability and embryonic mortality rate for broiler breeders post molting.

Item	Mating ratios (cock to hen)			SEM	*p*-value
	1:10	1:12	1:14		
Fertility rate, %	92.28^a^	92.01^a^	90.39^b^	1.27	0.037
Total hatchability, %	85.94^a^	84.89^a^	82.78^b^	1.26	0.002
Hatchability of fertile eggs, %	92.72	92.26	91.98	1.25	0.389
Early embryonic mortality rate, %	2.76	2.86	2.71	0.34	0.861
Late embryonic mortality rate, %	3.98	4.25	4.01	0.37	0.551
Total embryonic mortality rate, %	6.74	7.12	6.72	0.54	0.525

^a,b^Means within the same row with different superscripts differ significantly (*p* ≤ 0.05).

SEM: standard error of the mean.

Research has shown that a mating ratio of 1:10 resulted in the highest fertility levels for broiler breeders, as it combined a high degree of mating activity with relatively low levels of hostility and interference Casanovas and Wilson.^19^ However, in the case of a 1:14 mating ratio, some females may not have been mated due to increased sexual inactivity caused by forced molting, resulting in unfertile eggs. These findings align with Karousa et al.^20^, who stated that the reason for the improvement in fertility rate at lower mating ratio may be attributed to the fact that these groups’ female members received more mounting than those in other groups. According to Lambrechts et al.^21^, male stress may result from an overabundance of females, yet males with a reduced sex ratio can still mate successfully. Excessive male hostility and competition for territory and mating might negatively impact fertility when male numbers increase per female.^22^ Wishart and Staines^23^ in a previous study reported that among floor-mated broiler breeder hens, a specific subset of chickens that do not seem to be mating is the primary source of infertile eggs. Different cock reproductive capacities may be the cause of the observed variations in the number of fertile eggs and percent fertile obtained owing to different mating ratios.^24^

### Egg hatchability

It is important hatchery management should be aware of the variables that affect hatchability and chick quality to make better decisions. According to Fairchild et al.^25^, the key parameters that affect hatchability are egg fertility and embryonic mortality. In our study, total hatchability percentage significantly (*p* = 0.002) improved for breeders reared at mating ratios of 1:10 and 1:12 by 3.82% and 2.55% respectively, compared with those at 1:14 as shown in Table 2. However, there was no significant (*p* = 0.389) differences in hatchability of fertile eggs by mating ratio. Fertile eggs that were obtained from hens with the cock: hen ratios of 1:10 and 1: 12 hatched (92.28% and 92.01%, respectively) more than those from cock: hen ratio of 1:10 with a hatchability score of 90.39% as shown in Table 2. Understanding the underlying reason for low hatchability is crucial because improving low fertility can be achieved by managing the hens and cocks on the breeding farm well. Bobbo et al.^26^ found a positive correlation between egg fertility and hatchability. Additionally, Farooq et al.^27^ reported that during the incubation and hatching stages, hatchability can be influenced by fertility; when fertility declines, hatchability decreases as well. These findings suggest that a cock: hen ratio with a high percentage of fertile eggs will also give a high hatchability and this support our results, as mating ratios of 1:10 and 1:12 improved fertility rates and total hatchability without significant differences between them.

### Embryo mortality

Early embryonic mortality rate (*p* = 0.861), late embryonic mortality rate (*p* = 0.551) and total embryonic mortality rate (*p* = 0.525) were not affected by mating ratios (Table 2). It’s important to note that the early embryonic mortality rate is primarily associated with the condition of the breeder flock (such as nutrition and diseases), egg handling, and potential errors in hatchery procedures.^25^ These results align with previous research by by Ipek et al.^28^ and Alsobayel and Albadry^29^, who reported no significant differences in early, late, or total embryonic mortality for laying hens at different mating ratios. Furthermore, Atawalna and Agbehadzi^30^ indicated that no significant differences were observed in the hatchability of fertile eggs and embryonic mortality at different mating ratios for Guinea fowl.

## Conclusions

From the current results, the mating ratio in Arbor Acres Plus broiler breeders post molting did not affect egg production, chick weight, or embryonic mortality rate. However, the mating ratios 1:10 and 1:12 improved fertility rate and total hatchability. As a result, to reduce the cost of raising, it is recommended to use a cock: hen ratio of 1:12. Further studies on open-house farms and in large sample sizes must be conducted before applying these results more broadly.
